# Electron Beam Transparent Boron Doped Diamond Electrodes for Combined Electrochemistry—Transmission Electron Microscopy

**DOI:** 10.1021/acsmeasuresciau.2c00027

**Published:** 2022-07-14

**Authors:** Haytham E. M. Hussein, Georgia Wood, Daniel Houghton, Marc Walker, Yisong Han, Pei Zhao, Richard Beanland, Julie V. Macpherson

**Affiliations:** † Department of Chemistry, University of Warwick, Coventry CV4 7AL, U.K.; ‡ Diamond Science and Technology Centre for Doctoral Training, University of Warwick, Coventry CV4 7AL, U.K.; § Department of Physics, University of Warwick, Coventry CV4 7AL, U.K.

**Keywords:** boron doped diamond (BDD), transmission electron microscopy (TEM), BDD-TEM grids, atom resolution, identical location, electrocatalysis, electrodeposition, carbon corrosion, *in situ* heating TEM, manganese oxide, carbon TEM grids

## Abstract

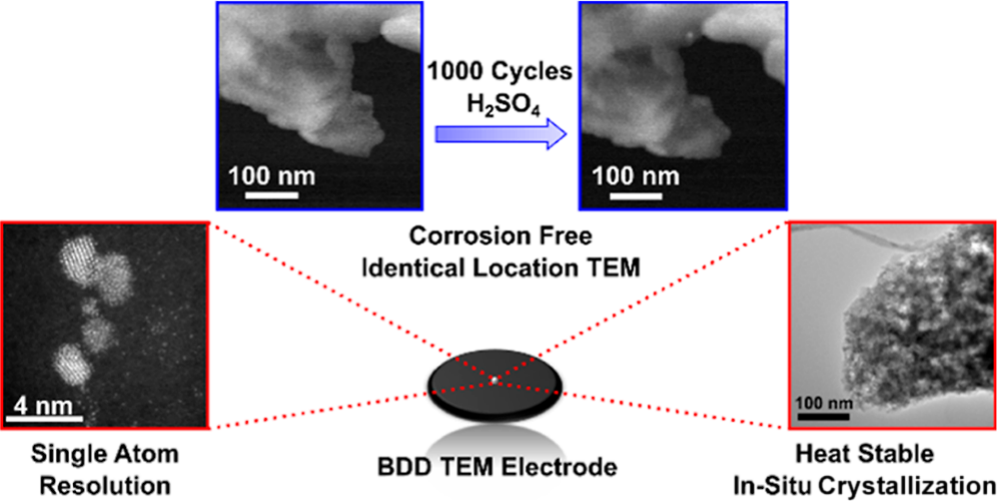

The majority of carbon based transmission electron microscopy (TEM) platforms (grids) have a significant sp^2^ carbon component. Here, we report a top down fabrication technique for producing freestanding, robust, electron beam transparent and conductive sp^3^ carbon substrates from boron doped diamond (BDD) using an ion milling/polishing process. X-ray photoelectron spectroscopy and electrochemical measurements reveal the sp^3^ carbon character and advantageous electrochemical properties of a BDD electrode are retained during the milling process. TEM diffraction studies show a dominant (110) crystallographic orientation. Compared with conventional carbon TEM films on metal supports, the BDD-TEM electrodes offer superior thermal, mechanical and electrochemical stability properties. For the latter, no carbon loss is observed over a wide electrochemical potential range (up to 1.80 V *vs* RHE) under prolonged testing times (5 h) in acid (comparable with accelerated stress testing protocols). This result also highlights the use of BDD as a corrosion free electrocatalyst TEM support for fundamental studies, and in practical energy conversion applications. High magnification TEM imaging demonstrates resolution of isolated, single atoms on the BDD-TEM electrode during electrodeposition, due to the low background electron scattering of the BDD surface. Given the high thermal conductivity and stability of the BDD-TEM electrodes, *in situ* monitoring of thermally induced morphological changes is also possible, shown here for the thermally induced crystallization of amorphous electrodeposited manganese oxide to the electrochemically active γ-phase.

## Introduction

Transmission electron microscopy (TEM) is a powerful analytical tool for characterizing nanomaterials at the atomic level.^1−3^ TEM can also be used in combination with electron diffraction, to solve crystal structures, and electron energy loss spectroscopy (EELS), to determine bonding and oxidation states.^4^ For the imaging of nanomaterials, a TEM grid coated with a continuous or partial thin film of an electron beam transparent material is often used. Thin films (3–30 nm) of carbon floated over a support substrate are particularly popular due to carbon being low electron scattering and electronically conductive. Here the carbon is typically an amorphous carbon film, although graphene oxide^5^ and graphene^6^ layers have also been used.

In electrocatalytic energy conversion applications carbon is also frequently used as the nanostructured electrocatalyst support for electrocatalytically active nanoparticles (NPs). The carbon, typically a carbon black, can take a variety of structures, which range in the ratio of sp^2^ to sp^3^ bonded carbon and the level of graphitization.^7^ Such carbons are used due to their low cost, electrical conductivity and reduced electrocatalytic activity towards the energy conversion processes of interest.^8^ To assess NP structure pre- and post-electrocatalysis, either the NPs or the NPs plus carbon black support, can be placed on the carbon TEM grid.^9,10^ Outside of energy applications, carbon film TEM grids have also found use in the elucidation of electrochemically driven NP deposition mechanisms.^11,12^

For *ex situ* studies, measurements are typically undertaken by dipping the TEM grid into an electrolyte solution, performing the electrochemical process of interest, removing from solution and then imaging the surface.^10,13^ This process typically requires the carbon film to be supported on a metal grid so an electrical contact can be made. Use of TEM “finder grids” (where the metal support contains a labeled co-ordinate grid) makes monitoring of the same location, pre- and post-electrochemical treatment much easier; this process is termed identical-location (IL-)TEM.^14−16^ However, interpretation of the current passed is challenging due to both the metal support and carbon film acting as an electrode. A few studies have overcome this issue by using bespoke holders which prevent electrolyte accessing the metal grid.^11,17,18^

More recently, scanning electrochemical cell microscopy (SECCM), where an electrolyte filled nanopipette is used to create a miniature electrochemical cell, has been employed with carbon film TEM grids.^19,20^ Here the SECCM tip was used to both electrodeposit NPs locally^19^ and measure the local electrocatalytic activity of pre-formed NPs.^20^ SECCM has also been used as a method for placing pre-formed NPs onto the working electrode for *in situ* electrochemical TEM observation.^21^

Whilst carbon is useful as an electrocatalyst support and TEM film it does have some drawbacks.^22^ The most notable one is the fact carbon can undergo oxidation and corrosion at potentials which are important for energy conversion reactions. These include, for example, during the start–stop cycle of a proton exchange oxygen reduction reaction (ORR) membrane fuel cell^23,24^ and at the potentials required for the oxygen evolution reaction.^25^ Corrosion of the carbon TEM film also complicates studies aimed at investigating electrocatalytically induced changes (morphological, chemical oxidation state) in NPs. Furthermore, for thermal studies, interactions between the TEM grid metal support and the nanostructure/carbon film should also be considered. For example, at elevated temperatures, certain metals *e.g.* nickel, can graphitize the carbon film,^26,27^ and metal atoms from the underlying support, can evaporate and redeposit, causing contamination.^28^

Given the above, it is interesting to consider the suitability of boron doped diamond (BDD) for correlative TEM-electrochemical (and thermal) measurements. BDD is an interesting alternative to sp^2^ carbon, as the sp^3^ carbon bonding results in an increased mechanical strength and corrosion resistance, both chemical and electrochemical. BDD is also an excellent conductor of heat.^29^ When used as an electrode material, in the more common oxygen terminated form, the surface properties are such that the double layer capacitance is low (<10 μF cm^–2^) and electrocatalytic processes such as ORR and water electrolysis are significantly kinetically retarded.^29^ Many of these properties make BDD an ideal NP electrocatalyst support.^30,31^ However, BDD has yet to be used routinely as a TEM electrode, due to the lack of methodologies available to fabricate BDD which is not only thin enough to be electron beam transparent (∼10 to 100 nm thick) but is also handleable. In this paper we describe a procedure to produce free standing (unsupported) BDD-TEM electrodes, and highlight their useful properties as corrosion free, temperature stable, TEM supports for applications of importance in the electrodeposition and energy conversion fields.

## Experimental Section

### Solutions and Materials

All solutions were prepared using Milli-Q ultrapure water with a resistivity of 18 MΩ cm (Millipore). (a) For acid cleaning, sulfuric acid (H_2_SO_4_ 95–97%, Scientific and Chemical Supplies Ltd.) and potassium nitrate (KNO_3_ 99.0%, Scientific and Chemical Supplies Ltd.) were used. (b) For metal electrodeposition, manganese oxide was deposited from a solution containing manganese chloride (MnCl_2_, 0.1 M, Sigma-Aldrich) and potassium chloride (KCl 99.0%, 0.1 M, Sigma-Aldrich) acidified with hydrochloric acid (HCl 37%, 0.01 M, Sigma-Aldrich). (c) For electrochemical characterization, hexaamineruthenium(III) chloride [Ru(NH_3_)_6_Cl_3_ 99%, Strem Chemicals] was employed as a redox couple and potassium nitrate (KNO_3_ 99%, Sigma-Aldrich) as supporting electrolyte. (d) Sulfuric acid (H_2_SO_4_ 95–97%, Scientific and Chemical Supplies Ltd.) was used for long term electrochemical stability testing. Where commercial TEM grids were used for comparison, carbon films on a 300 mesh copper or gold support (Agar Scientific Ltd., UK) were employed.

All BDD was provided by Element Six Ltd., Oxford, UK, and was grown using microwave chemical vapor deposition (CVD) to a suitable thickness so that it could be removed from the non-diamond growth support.^32^ The material was suitably doped with boron (>10^20^ B atoms cm^–3^) such that the material was above the metallic threshold.^29,32^ Both surfaces were mechanically (resin bonded) polished, to thin the material to ∼50–80 μm and produce surfaces of ∼nm surface roughness.^33^ Laser micromachining of the BDD was carried out using a 355 nm Nd:YAG 34 ns laser (Oxford Lasers) to cut out disks of 3 mm diameter. To remove machining debris the electrodes were acid cleaned by immersing in concentrated H_2_SO_4_ (saturated with KNO_3_) at ∼200 °C, for 30 min, followed by rinsing with ultrapure water before cleaning for 30 min in concentrated H_2_SO_4_ only, at ∼200 °C.^34^

Argon ion milling and polishing of the BDD disk was carried out using a GATAN precision ion polishing system (PIPS). The BDD was mounted on a post support using glycol-phthalate bonding wax (Agar Scientific), allowing continuous milling as the sample rotated. First one side was milled for 2.5 h, then the disk was turned over and the other side milled for 2 h and then in 15 min intervals until light transmission through the center of the grid was visible *i.e.* a hole had formed. To reduce surface roughness the disk was mounted in a clamp support for a final low energy polish of both sides of the disk simultaneously. This was achieved using a modulated ion beam at a lower accelerating voltage and angle of incidence, for 30 min. To increase the robustness of the Ohmic contact to BDD, a small segment of the upper portion of the disc was laser roughened (532 nm Nd:YAG 15 ns laser).^34^ The disk was again acid cleaned (*vide supra*) and electrical contact made to this lasered region by either sputtering a Ti (10 nm)/Au (400 nm) contact (Moorfield MiniLab 060 Platform), followed by annealing for 5 h at 400 °C,^32^ or by application of a conductive carbon ink (MG Chemicals, 838AR). Note, as a result of laser roughening and acid cleaning, the small region of the BDD onto which the contacts are placed, contains a very thin surface layer of non-diamond carbon.^34^ This is especially useful when making an Ohmic contact using the carbon ink.

### Electrochemical Measurements

Cyclic voltammetry (CV) was carried out using a three-electrode setup controlled by a potentiostat (Ivium CompactStat, Holland) with a saturated calomel electrode (SCE; ALS, Japan) or an Ag|AgCl electrode (non-leak, ∼3.5 M KCl, WPI) used as the reference and a Pt coil as the counter. Electrical contact to the BDD-TEM electrode was made using a metal clamp. For electrochemical measurements the BDD-TEM electrode was dipped into the electrolyte, ensuring that the central hole was fully immersed in solution and the electrical contact remained dry.^33^ After any electrochemical process and before imaging, the BDD-TEM electrode was rinsed by gently dipping in ultrapure water and then left to dry in a desiccator, held under vacuum.

For electrochemical characterization, a three-electrode droplet cell set-up was used^35^ with a 1 mm diameter disk on the BDD-TEM electrode exposed using Kapton tape. A 200 μL droplet of electrolyte solution was placed on the electrode surface for each measurement. Solvent window and capacitance measurements were run in 0.1 M KNO_3_ at a scan rate of 0.1 V s^–1^. The electrode response for the redox couple Ru(NH_3_)_6_^3+/2+^ was investigated by recording CVs of 1 mM Ru(NH_3_)_6_Cl_3_ in 0.1 M KNO_3_ at a scan rate of 0.1 V s^–1^ with a step size of 1 mV. Solvent windows were defined for a geometric current density of ±0.4 mA cm^–2^.^32^

Surface roughness measurements were made using both an atomic force microscope (AFM, Innova, Bruker, USA) and a white light interferometer (WLI, Bruker ContourGT Bruker Nano Inc., USA). Image analysis was performed using Gwyddion 2.5.2.^36^ Contact angle measurements were recorded using a drop shape analyzer (DSA100E, Krüss Scientific, Germany) with a water droplet of volume 50 μL. Measurements were recorded in triplicate, with the surface dried carefully in between using a lint free tissue.

Field emission scanning EM (FE-SEM) was used to image the BDD-TEM electrode. Images were recorded using the in-lens, secondary electron (SE2), and scanning TEM (STEM) detectors on a Zeiss Gemini FE-SEM 500 operating at 20 kV. TEM imaging and electron diffraction on the BDD-TEM electrodes were carried out using a JEOL JEM 2100 (LaB_6_) TEM at 200 kV. *In situ* TEM heating was also achieved in this TEM with a double tilt heating holder (model 652, Gatan Inc., US). Atom resolution annular dark field (ADF) images were recorded in a double aberration-corrected JEOL JEM-ARM200F operated at 200 kV.

For long term electrochemical stability testing in acidic media the coastline around the hole edge of a BDD-TEM electrode was mapped using the double-corrected JEOL JEM-ARM 200F TEM, operated at 200 kV. Multiple areas were selected and ADF images taken and compared before and after electrochemical cycling. To estimate the change in thickness of the BDD-TEM electrode EELS spectra were collected in STEM mode, at a probe convergence semi-angle of 32 mrad, a spectrometer semi-collection angle of 25 mrad, and a dispersion of 0.25 eV per channel. The energy resolution of the EELS measurements was 1.8 eV, as estimated from the full-width-half-maximum of the zero-loss peaks.

X-ray photoelectron spectroscopy (XPS) was conducted using a Kratos Analytical Axis Ultra DLD spectrometer with a monochromated Al Kα X-ray source (1486.69 eV) in a chamber with a base pressure below 1 × 10^–10^ mbar. Samples were mounted on the sample bar using electrically conductive carbon tape. High resolution C 1s spectra were collected using a pass energy of 20 eV (resolution of approximately 0.4 eV). Data from the BDD-TEM electrodes were collected using an analysis area of 55 μm diameter, to probe as close to the hole edge as possible. For the control sample (mechanically polished BDD) the data were acquired using a spot size of 110 μm in order to increase the overall count rate. C 1s spectra were obtained using take-off angles of 90 and 30° with respect to the surface plane. To investigate the different carbon chemical environments at the electrode surface, all data collected were fitted in CasaXPS using Lorentzian–Gaussian lineshapes and Shirley backgrounds, with asymmetry included for the sp^2^ bonded carbon C–C peak.

## Results and Discussion

### BDD-TEM Electrode Fabrication

**Figure 1:**
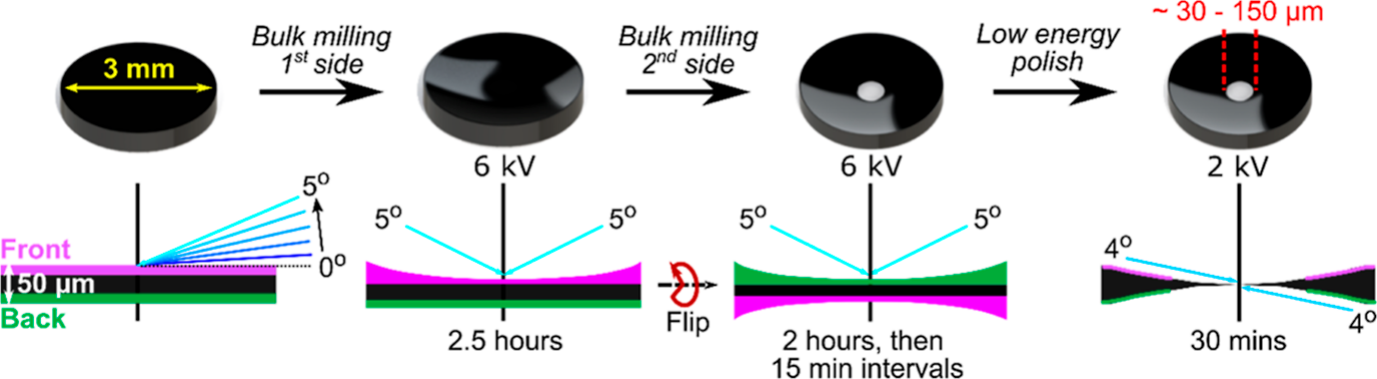
Schematic illustration of the PIPS milling/polishing procedure to thin the center of the BDD disk to electron beam transparency. The black disks represent top down schematics, whilst the below images represent side profiles. 0–5° represents ion beam angle of incidence relative to the surface. Note the hole is not to scale and is significantly smaller than shown.

Compared to non-diamond carbons, achieving electron beam transparent diamond electrodes is challenging, due to the limited number of growth methods. Here, a top down approach was employed where the starting point was the production of freestanding and double-sided polished polycrystalline BDD electrodes, as thin as possible, but still able to be handled easily. For this reason 50–80 μm thick BDD electrodes were produced by CVD growth with subsequent mechanical polishing, and then cut into disks of 3 mm diameter to make them suitable for insertion into the TEM holder. To achieve electron beam transparency the electrode was first argon ion milled, on each face, and then the energy of milling lowered to ion “polish” both surfaces simultaneously as depicted schematically in Figure 1. Note as the surface thins most in the central region, a concave surface results. The outer edges of the BDD-TEM electrode remain at the starting thickness, ensuring ease of handling.

Initially the highest accelerating voltage was used for the fastest milling rate to remove the bulk of the BDD. An angle of incidence in the middle of the accessible range was employed. If the angle was set too high, material was removed more quickly, and a smaller region of electron beam transparent material resulted. A middle value was found to be a time efficient compromise. The exact milling time was dependent on the starting material thickness. As Figure 1 shows, one face of the disk was milled (here using 6 keV and an angle of 5° rotating at 0.1 rpm). The sample was then flipped, and the other side milled using the same parameters until a small hole (*ca.* 30–150 μm in diameter) formed in the center of the BDD disk. The observation of a hole indicates the presence of a region of BDD that is thin enough to be electron beam transparent around the hole edge. Typically, if the hole is increased in size smaller areas of electron beam transparency result. For nine BDD-TEM electrodes examined we found an approximately linear relationship (*R*^2^ = 0.98) between the diameter of the hole and the width of the electron beam transparent area. As milling results in ripples (*vide infra*) on the BDD surface, a final lower energy ion polish (1–2 keV) was required (Figure 1) at a slightly lower angle to reduce the surface roughness whilst avoiding further increasing the size of the hole and reducing the electron transparent area. It should be noted that polishing does not remove the ripples completely but does reduce ripple amplitude. A troubleshooting guide is presented in Supporting Information 1, Table S1 for fabrication of BDD-TEM electrodes *via* this method. An image of the resulting BDD-TEM electrode being handled by tweezers is also presented (Figure S1).

To apply an electrical contact to the BDD-TEM electrode, two approaches were investigated: a Ti/Au contact,^37^ and a conductive carbon ink contact (see Experimental Section). To assess the impact of both contacts on electrochemical performance, the uncompensated resistance, *R*_u_, was measured in a solution containing 0.1 M KNO_3_. Very similar areas were immersed (∼0.11 cm^2^) and the distance between the BDD-TEM electrode and reference electrode was kept constant.^38^ For both electrodes, similar *R*_u_ values of 229 ± 1 Ω (Ti/Au contact) and 346 ± 4 Ω (carbon ink contact) were obtained (see Supporting Information 2), indicating both approaches were valid. Whilst Ti/Au is commonly used to make an Ohmic electrical contact to BDD,^32^ it does require access to a sputter system and care is required during sputtering to prevent Au spill over onto the BDD-TEM electrode due to its concave profile. In contrast, the carbon ink can be painted onto the laser roughened (and non-diamond carbon containing) area of the BDD surface by hand. This method thus represents a cheaper, quicker, metal contamination free option, particularly useful for long-term electrochemical experiments.

### Surface Characterization

**Figure 2:**
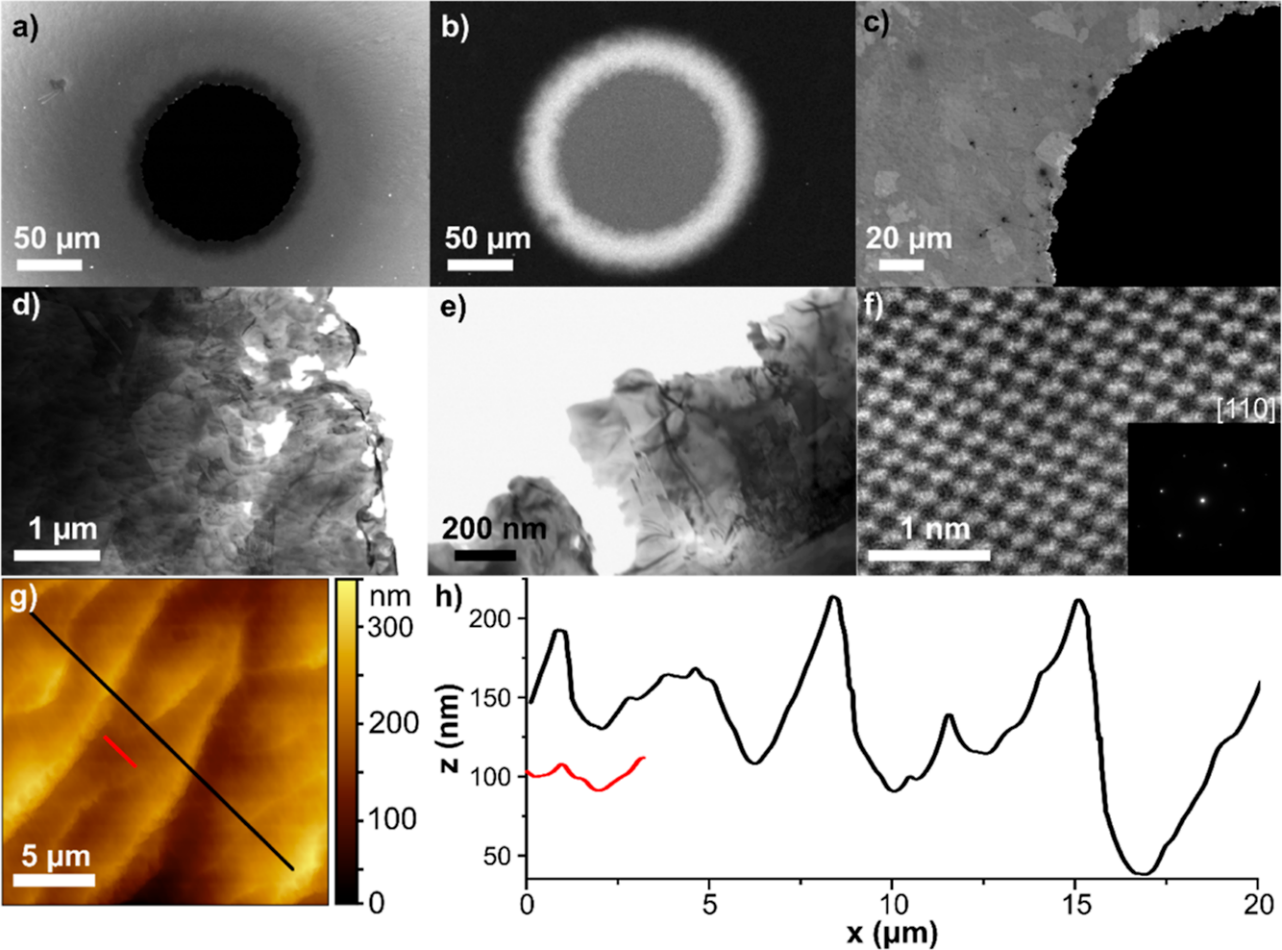
FE-SEM (a–c) and STEM (d,e) images of a typical BDD-TEM electrode recorded at 20 kV using the (a,c) in-lens, (b) SE2, and (d,e) STEM detectors. (f) ADF-STEM image taken from a grain comprising the BDD-TEM substrate, inset: electron diffraction pattern showing a (110) crystallographic orientation of the surface. (g) 20 × 20 μm tapping mode AFM topography image (0.25 Hz) of a BDD-TEM electrode after PIPS milling and polishing, with (h) corresponding surface line profiles, where *z* is the measured height and *x* is the position along the red and black lines marked in (g).

FE-SEM and STEM images of a typical BDD-TEM electrode fabricated *via* the method outlined are shown in Figure 2, focused in on the region around the central hole. The hole in Figure 2a is ∼130 μm in diameter. The region of electron beam transparency appears as a dark ring in Figure 2a (in-lens image) and more clearly as a bright ring in Figure 2b (SE2 image), *ca.* 50 μm in width (at 20 kV). Figure 2c shows the polycrystalline structure of the BDD, and the uneven coastline of the hole, the latter which is essential for IL-TEM. Figure 2d,e show higher resolution STEM images of the very edge of the hole. In Figure 2d, sub-micron sized holes have also formed at the very edge. Such features were often observed when the BDD was very thin. For TEM analysis, the thinner the BDD support the higher the contrast resolution of the nanostructure of interest, as scattering from the BDD background is reduced. Figure 2f shows an ADF-STEM image of the atomically resolved structure of the BDD-TEM electrode. Selective aperture electron diffraction (SAED) (inset to Figure 2f) reveals a (110) crystallographic orientation of the diamond grain, which is observed universally over the surface, highlighting the dominant (110)-texture of this polycrystalline surface.^33,39^

The topography of the BDD-TEM electrode was investigated using AFM, Figure 2g,h and WLI (Supporting Information 3, Figure S3). The AFM measurements (Figure 2g) were recorded very close to the hole edge. Whilst WLI has the advantage of accessing larger areas than AFM it does show a significantly reduced *x*, *y* spatial resolution. Figure S2a(i) shows a WLI image of the central region of the BDD-TEM electrode covering a 1.2 mm diameter region. Figure S3a(ii) shows the corresponding *x*, *y* topography profile from the hole edge to 0.6 mm away [red line in Figure S3a(i)] highlighting the increasing non-linear thickness of the BDD moving away from the hole. The ripples in the surface topography are evident in the AFM images of topography (Figure 2g) and in higher magnification WLI measurements [Figure S3b(i)]. Such ripples have also been seen when diamond surfaces have undergone ion bombardment.^40^ The mechanism of ripple formation is still disputed despite being first observed over twenty years ago.^40,41^ The RMS roughness was calculated to be 48.3 nm across the whole area in Figure 2g, whilst the 1-dimensional RMS roughness, across the black line in Figure 2g, is 4.7 nm (Figure 2h). If the line profile is focused in a featureless region of the AFM image (red line in Figure 2g), the roughness is reduced to 1.5 nm. All data presented herein were obtained using disks milled using a PIPS I system. A PIPS II ion milling system (which operates at higher current densities) can be used to prepare comparable electrodes, see Supporting Information 3, Figure S4 for FE-SEM and AFM characterization.

**Figure 3:**
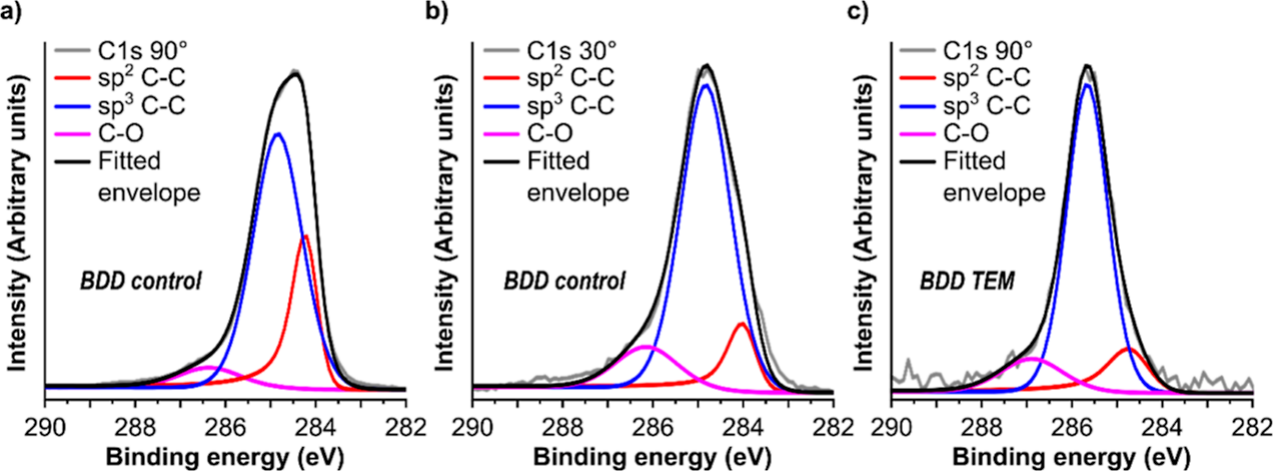
(a–c) Fitted C 1s XPS spectra of (a) un-milled, mechanically polished BDD (BDD control) at 90° take-off angle, (b) BDD control at 30° take-off angle and (c) ion milled BDD-TEM electrode measured 55 μm away from the edge of hole (BDD TEM) at 90° take-off angle. Note a smaller spot size for (c) is contributing to the increased noise on the signal.

To assess the impact of ion milling on the surface chemistry of the BDD-TEM electrodes, XPS was employed, Figure 3, at room temperature. Comparison measurements were made with a mechanically polished surface (the starting surface pre-ion milling), Figure 3a,b, herein referred to as the BDD control. Both surfaces were acid cleaned prior to XPS analysis. The fitting of the C 1s spectra of the BDD control (Figure 3a, take-off angle 90°) indicates a sp^3^ C–C/C–H contribution of approximately 72%, with a sp^2^ C–C character of 21% (Supporting Information 4, Table S3). The C–O contribution is approximately 7%.^39^ Higher order oxides (*e.g.* O=C–O) which occur at >288 eV, make a minimal contribution (<1% of the fitted peak). In order to provide greater surface sensitivity, the XPS take-off angle was decreased to 30°, to reduce the sampling depth by a factor of two.^42,43^ Using the inelastic mean free path calculator (IMFP-TPP2M)^44^ the penetration depth (for C 1s) reduces from 9.9 to 4.5 nm. Under the more surface-sensitive conditions at 30°, the main difference is a reduction in the sp^2^ carbon component to 9% of the fitted envelope. This result indicates that a significant component of the sp^2^ carbon signal in Figure 3a is likely to be sub-surface, which is not surprising as mechanical polishing of diamond is known to induce sub-surface damage.^45^ There is also an increase in the C–O contribution from ∼7% at 90° to ∼13% at 30°, as a result of the lower collection angle being more surface sensitive; the O termination is only found at the surface of the BDD. Adventitious carbon signals are minimal.

When the 90° take-off angle BDD control data (Figure 3a) is compared to the 90° take-off angle BDD-TEM XPS spectrum (Figure 3c), the main difference is a clear decrease in the sp^2^ carbon character from 21 to 10% for the BDD-TEM electrode. The value is now close to that seen for the 30° take-off angle BDD control data. This relative reduction in the sp^2^ carbon content in the more surface-specific geometry confirms the presence of sub-surface damage from the initial mechanical polish, which is removed during the ion milling and ion polishing process. The data also indicates no subsequent ion milling induced sub-surface sp^2^ carbon creation. Table S3, Supporting Information 4, gives fittings of the C 1s data for both electrodes expressed as percentages of the total fitted envelope. There is a shift of approximately 0.9 eV in the absolute binding energy of the C 1s peak, and thus of the binding energies of each assigned peak in the fitting, for the BDD TEM electrode when compared to the BDD control electrode. The reason for this shift is unknown. To account for this, binding energies have been considered relative to the assigned sp^3^ carbon peak for each electrode (Supporting Information 4, Table S3).

Comparison XPS measurements were also obtained on a commercial thin film carbon Au backed (C/Au) TEM grid (Supporting Information 4, Figure S5 and Table S4) at room temperature and at a take-off angle of 90°. The sp^2^ and sp^3^ carbon content was found to be 62 and 27% respectively, in similar proportions to fittings reported in the literature.^20^ As expected with an increased sp^2^ carbon content, more significant contributions from C=O, O=C–O and π–π* (2% of the envelope each) were observed. Contact angle measurements were recorded to compare the hydrophobicity and wetting of a BDD-TEM electrode *versus* a commercial amorphous carbon coated TEM grid (Supporting Information 5, Figure S6). For the BDD-TEM electrode, the disk was only ion milled on one side to prevent formation of the hole which would adversely affect the observed wetting. Contact angles of 62.2 ± 0.5° (BDD-TEM) and 83.6 ± 1.1° (amorphous C film) were measured showing that the BDD-TEM electrode is more hydrophilic than the carbon film. This is also advantageous for TEM electrode aqueous based applications, where uniform wetting of the TEM electrode is preferred. The value recorded for the BDD-TEM electrode is at the upper end of those recorded on other oxygenated BDD surfaces.^29^

### Electrochemical Measurements

#### Electrochemical Characteristics

To further investigate the properties of the ion milled/polished surface compared with a mechanically polished control electrode, electrochemical characterization of the solvent window and electrical double layer capacitance was carried out. Solvent window, double layer capacitance and CV peak to peak separation data (Δ*E*_p_) for the redox couple Ru(NH_3_)_6_^3+/2+^ are shown in Supporting Information 6, Figure S7. Wide and featureless solvent windows with values of 3.2 and 3.5 V, capacitance values of 5.3 and 4.3 μF cm^–2^, and Δ*E*_p_ values of 70 and 68 mV were obtained for the ion milled/polished BDD-TEM and BDD control (mechanically polished) electrodes, respectively. The responses for all three parameters for the two differently prepared electrode surfaces are similar with the ion milled/polished surface showing only a small decrease in solvent window, and a very slight increase in both capacitance and Δ*E*_p_ compared to the control material.

#### Electrochemical Corrosion Properties

**Figure 4:**
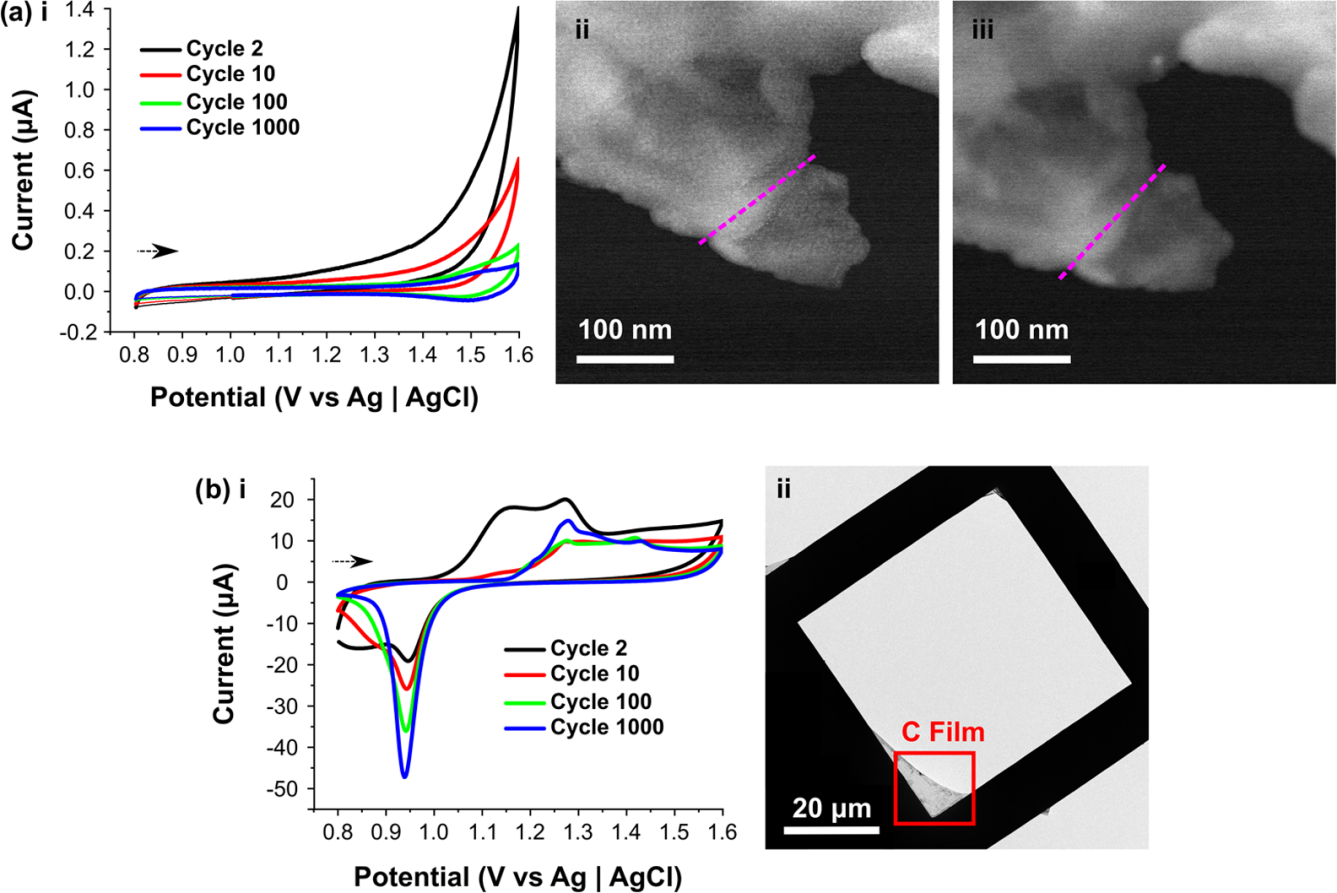
Electrochemical AST on (a) BDD-TEM electrode and (b) commercial C/Au TEM grid. [a(i)] CV response for cycles 2, 10, 100, 1000. [a(ii,iii)] High magnification IL-STEM ADF images of the BDD-TEM electrode [a(ii)] before and [a(iii)] after 1000 cycles. Dashed purple lines indicate where EELS line profiles were taken. [b(i)] CV response for cycles 2, 10, 100, 1000. [b(ii)] Representative low magnification TEM image of the commercial C/Au TEM grid after AST, showing significant damage; very little of the film remains (red box). AST was carried out from 0.8 to 1.60 V *vs* Ag|AgCl for a total of 1000 cycles, in 0.5 M H_2_SO_4_, at a scan rate of 0.1 V s^–1^. Black arrows indicate initial scan direction.

To assess the susceptibility of the BDD-TEM electrode to electrochemical corrosion that is carbon dissolution, the electrode was subjected to accelerated stress testing (AST),^24^Figure 4a(i–iii). AST experiments are typically used in electrocatalysis degradation and carbon corrosion support testing and are reflective of the extremes experienced in practical energy conversion systems. Specifically, this experiment involved cycling in sulfuric acid (0.5 M H_2_SO_4_) for 1000 CV cycles at 0.1 V s^–1^ (total experimental run time = 5 h) over the potential range 0.80 to 1.60 V *versus* Ag|AgCl (the maximum equivalent to 1.82 V *vs* RHE).^24^ These conditions reflect the extreme positive potentials a proton exchange membrane fuel cell experiences^24^ and the typical operational voltages of acid based electrolyzers.^46^ Here the experiment was carried out with both a BDD-TEM electrode and C/Au TEM grid dipped into solution. A similar immersion depth was used for both (∼2 mm). For the former, a carbon conducting ink was used to create the electrical contact. This avoids Au dissolution from a Ti/Au contact and potential re-deposition (contamination) on other areas of the electrode, as the sulfuric acid will evaporate during the long timescale of this experiment.

Figure 4a(i) shows the CV response on the BDD-TEM electrode over 1000 cycles. Over the first 10 cycles there is a change in the current magnitude, where the currents at the more extreme positive potentials decrease with increasing scan number. We attribute this initial behavior to an electrochemical cleaning of the BDD surface. This cleaning continues, decreasing with cycle number up to 100 cycles, where a stable response is observed, the peak current at 1.60 V changes slowly: less than 100 nA decrease over the remaining 900 cycles. The observed stable response and the very low currents passed for the electrode area immersed (∼2 mm), reflect the electrochemical stability of the BDD-TEM electrode in this AST potential scan range.

To verify the absence of dissolution (corrosion) of the BDD electrode IL-STEM EELS measurements were carried out around the hole edge. IL-EELS can quantify thickness changes in the same area of the electron beam transparent BDD in response to the AST cycling treatment. Figure 4a(ii,iii) show representative high magnification ADF-STEM images of the BDD-TEM electrode hole edge recorded before [Figure 4a(ii)] and after [Figure 4a(iii)] AST. Identical location imaging on the BDD-TEM electrode was possible by finding unique and recognizable features around the hole edge (Supporting Information 7). EELS spectra (pixel size = 3 nm) were acquired across all areas of the BDD electrode BDD area shown in Figure 4a(ii,iii). The purple lines indicate the specific line profiles across which EELS thickness measurements were taken as shown in Supporting Information 7, Figure S8. For each pixel within the EELS spectrum image the BDD thickness, *t*, was measured using the absolute log ratio method,^47^ (eq 1)
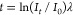
1λ is the calculated inelastic mean free path of 200 kV electrons in diamond (=97.61 nm^48^), *I*_0_ is the area under the zero loss peak and *I*_*t*_ is the total area under the whole spectrum. Across the lines shown in Figure 4a(ii,iii), EELS analysis gave an average *t* of 20.4 nm before cycling and 21.7 nm after (28 points per line, Supporting Information 7, Figure S9). The difference in these values is within the experimental error of the calculation, which is estimated to be *ca.* 5%,^47^ and indicates the BDD is not electrochemically corroding during AST. This is also in agreement with no observed change in the shape of the coastline.

Figure 4b(i) shows the CV response of the commercial C/Au TEM grid subject to the same AST. Clear differences are observed compared to the BDD-TEM electrode [Figure 4a(i)]. Firstly, the current responses are significantly larger, and the CV response changes rapidly over the first 10 cycles [Figure 4b(i)]. Observed is a rapidly diminishing peak at 1.10 V, attributed to surface oxidation and corrosion of the C film. Thermodynamically C-oxidation can occur at potentials as low as ∼0 V *versus* Ag|AgCl,^24^ although in practice it is kinetically limited, occurring at much higher potentials.^25^ With increasing scan number, growth of the reductive peak at 0.90 V *versus* Ag|AgCl is also observed. This peak is attributed to cathodic stripping of Au surface oxides (AuO_*x*_),^49^ formed during the oxidative part of the scan. The Au support, also exposed to solution, is converted to AuO_*x*_ at electrode potentials >∼1.2 V *versus* Ag|AgCl. The increasing AuO_*x*_ response with increasing number of cycles also indicates corrosion of the thin C film coating the top surface of the Au grid, which results in more Au being exposed to solution with time. Unlike BDD, the C/Au TEM grid CV response is still changing after 100 cycles albeit less significantly than during the initial 10 cycles, and by the 1000th cycle a response close to bulk gold is observed.^49^ This indicates that a significant amount of carbon has been removed or damaged. The extent of corrosion damage to the carbon film was confirmed by TEM imaging. Figure 4b(ii) shows the majority of the film is no longer present, only a small region of the carbon film remains, in a zone close to the bottom edge. This data highlights the usefulness of BDD-TEM electrodes as corrosion-free support substrates for TEM investigations of electrocatalyst stability under AST conditions.

### Electrochemical Deposition of Metals and Metal Oxides

**Figure 5:**
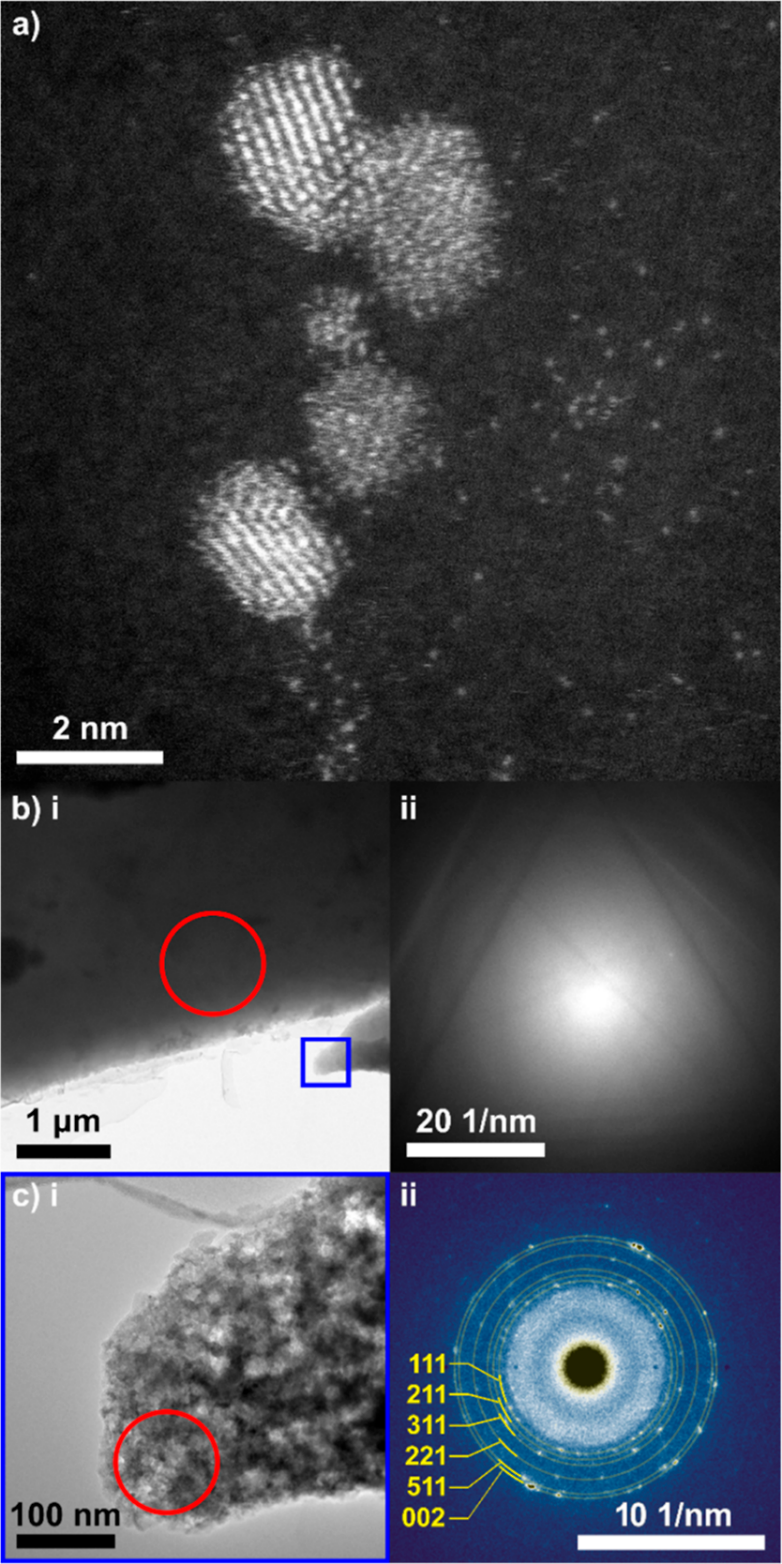
(a) High magnification STEM image of electrodeposited Au single atoms and NPs. TEM images and corresponding diffraction patterns of MnO_2_ electrodeposited on a BDD-TEM electrode (b) before heating and (c) after heating to 400 °C. Red circles in (i) indicate the areas that diffraction patterns shown in (ii), false color map adopted, were recorded over. Blue square in [b(i)] indicates the region magnified in [c(i)].

To highlight the capability of the BDD-TEM electrode as a combined electrodeposition and TEM imaging platform, Au nanostructures were electrodeposited on the electrode from a solution containing 1 mM [AuCl_4_]^−^ in 0.1 M HClO_4_. A high driving potential of −0.5 V *versus* SCE was employed for a very short period of time, 10 ms, to minimize the size of the nanostructures electrodeposited. As can be seen under these conditions observation of both crystalline Au NPs and isolated single Au atoms is possible, Figure 5a. Being able to image an isolated atom in TEM also brings the advantage of using the associated intensity signal to quantify the number of atoms in an isolated nanostructure.^33,50^ Given the mechanical and chemical robustness of the BDD-TEM supports, repeated imaging in the same location is also possible (IL-TEM),^33^ as shown in Figure 4a(ii,iii).

Diamond also has the advantage it is thermally stable up to *ca.* 1500 °C in vacuum and 950 °C in air^51^ and is an excellent conductor of heat^51,52^ (∼700 W m^–1^ K^–1^ at 300 K). These properties not only enable BDD-TEM substrates to be used in high temperature electrochemical applications,^53^ they also ensure that the temperature the BDD-TEM electrode experiences during *in-situ* TEM heating, matches that of the TEM heater. Here, BDD-TEM electrodes were employed to investigate the temperature-induced crystallization of electrodeposited amorphous manganese oxide (MnO_2_). MnO_2_ is used commercially in alkaline batteries due to the low cost and natural abundance of Mn.^54^ The electrochemically active γ-phase is favored due to its ability to facilitate proton intercalation.^55,56^

Figure 5b(i) shows a TEM image of electrodeposited MnO_2_ on the BDD electrode. The interface between light and dark regions in the image indicates the BDD-TEM hole edge. Deposition was achieved by applying a potential of 1.50 V *versus* SCE for 50 s in 0.1 M MnCl_2_ with 0.1 M KCl as a supporting electrolyte, acidified with HCl (0.01 M).^57^ The electrode was then dipped in distilled water to remove any salt residues and left to air dry. At this potential and pH, Mn^2+^ is first oxidized to Mn^3+^, followed by acid-catalyzed hydrolysis to MnO_2_. Note, electrodeposition on the BDD-TEM electrode was carried out *ex situ* prior to placement in the TEM for the subsequent imaging/heating experiments. SAED data of the MnO_2_, recorded in the region of the red circle in Figure 5b(i), indicates that the electrodeposited MnO_2_ is amorphous due to the diffuse ring in Figure 5b(ii). To determine the thermal conditions (temperature/heating time) which induce crystallization of this material and identify the resulting phase, the MnO_2_-BDD-TEM electrode was heated *in situ* in the TEM and under vacuum. The electrode was heated first to 50 °C, then allowed to cool to ambient temperature (in the TEM) and an image/diffraction pattern taken. This process was repeated, with the heating temperature increased in 50 °C increments (*i.e.* 50, 100, 150 °C *etc.*), until crystallization was observed due to the emergence of a diffraction pattern. Upon reaching 400 °C, TEM [Figure 5c(i)] and the corresponding SAED [Figure 5c(ii)] showed transformation to the crystalline form. Usefully, diffraction from the crystalline BDD electrode itself also allowed accurate calibration of the camera length (Supporting Information 8, Figure S10). Latticespacingd* values were measured from the SAED pattern [Figure 5c(ii)] allowing the deposit to be identified as the electrochemically active γ-MnO_2_ (Supporting Information 8, Table S5),^56^ the preferred phase of MnO_2_ for battery applications.

For some studies, it may be of interest for the nanostructured material to undergo heating in air, with TEM imaging taking place before and after. To assess the suitability of BDD-TEM electrodes under these conditions, experiments were conducted where both BDD-TEM and commercial C/Cu grids were heated to 200 °C and then 400 °C in air for 4 h each. Visual inspection of the grids using an optical microscope was carried out. For the BDD-TEM electrode no visual changes were observed. In contrast, for C/Cu the same grid could not be used throughout due to visible damage to the grid after the first heating experiment, Supporting Information 9, Figure S11. In particular, holes were seen forming due to thermal oxidation of the carbon film,^58^ which increased in severity with time, in addition to bending of the grid. This further emphasizes the wider range of operating/experimental conditions accessible when using a BDD-TEM electrode.

## Conclusion

A top down facile fabrication method for the production of re-useable, electron beam transparent and electrically conductive BDD-TEM electrodes, using argon ion milling and polishing of thin (<100 μm) BDD has been demonstrated. In contrast with conventional C TEM grids, which typically require a metal support for handling, the resulting BDD-TEM electrodes self-support. This was due to the non-uniform nature of the milling process where the central region was thinned the most, resulting in a concave profile to both sides of the BDD-TEM grid and a very small hole (typically 50–80 μm) in the center. For combined electrochemical-TEM measurements this also meant the electrochemical response was only due to the BDD and not a combination of BDD and metal support. The electron beam transparent regions of the BDD-TEM electrode were shown to be thin enough to facilitate resolution of electrochemically deposited and isolated single atoms (of Au). The central hole was also extremely helpful for IL-(S)TEM experiments, where the distinct shape of the hole edge was used to locate specific areas for repeat imaging.

A combination of techniques: SEM, TEM (including EELS), WLI, AFM and XPS, contact angle and electrochemical were employed to characterize the surface and investigate the impact of ion milling on the BDD surface and electrochemistry. XPS determined the BDD kept its sp^3^ carbon character with minimal surface damage after ion milling. TEM diffraction studies showed a dominant (110) crystallography of the surface. Useful electrochemical properties in terms of large aqueous solvent window and low double layer capacitance, were also retained. Moreover, the BDD-TEM grid was shown to be chemically and electrochemically resistant to carbon corrosion when subject to AST in acid, unlike commercial C/metal TEM grids. Such properties are extremely useful when developing NP catalyst supports, and are particularly suitable for correlative electrochemical-TEM experiments, under corrosion free support conditions. Thermal stability in air was evaluated (and compared against commercial C/metal grids) by heating up to 400 °C. The BDD-TEM electrodes remained intact unlike the C/metal grids. The advantage of having a thermally stable and electrically conductive TEM grid was highlighted by using the BDD-TEM electrodes in combination with *in situ* TEM heating to investigate the temperature of crystallization for electrodeposited amorphous MnO_2_. Transition to the electrochemically-active γ-MnO_2_ phase was shown at 400 °C.

Finally, with the current design of BDD-TEM electrode, the center of the disk contains a very small hole. The mass transport profile at the very edge of the hole will be different to that further away. Future work is focused on the development of BDD-TEM electrodes which are hole free and where the whole surface is electron beam transparent. Such electrodes should also find promise for combined optical detection-electrochemical measurements.

## Supporting Information Available

Supporting Information 1. Troubleshooting guide; 2. Uncompensated resistance measurements of BDD-TEM electrodes; 3. Additional surface characterization data; 4. Additional XPS data; 5. Contact angle measurements; 6. Electrochemical characterization; 7. EELS data; 8. MnO_2_ crystallization *d*-spacing data; 9. TEM grid temperature stability in air (PDF)

## Notes

All raw data used in this manuscript can be found in the Warwick Research Archive Portal http://wrap.warwick.ac.uk/167219.
