# Supplementary material for: Electron Beam Transparent Boron Doped Diamond Electrodes for Combined Electrochemistry—Transmission Electron Microscopy
Source: ACS Meas Sci Au. 2022 Jul 14;2(5):439–48. doi: 10.1021/acsmeasuresciau.2c00027 (PMC9585633; doi:10.1021/acsmeasuresciau.2c00027)
Supplement: Supplementary file 1 — tg2c00027_si_001.pdf [file tg2c00027_si_001.pdf]

Electronic Supporting Information

Electron Beam Transparent Boron Doped Diamond Electrodes for Combined Electrochemistry – Transmission Electron Microscopy

Haytham E. M. Hussein,[a‡] Georgia Wood,[ab‡] Daniel Houghton,[a‡] Pei Zhao,[a] Marc Walker,[c] Yisong Han,[c] Richard Beanland,[c] and Julie V. Macpherson[a]*

[‡] authors made equal contributions

[a] Department of Chemistry, University of Warwick, Coventry, CV4 7AL, UK

[b] Diamond Science and Technology Centre for Doctoral Training, University of Warwick, Coventry, CV4 7AL, UK

[c] Department of Physics, University of Warwick, Coventry, CV4 7AL, UK

*Corresponding author: j.macpherson@warwick.ac.uk

Table of Contents

ESI 1: Troubleshooting guide

ESI 2: Uncompensated resistance measurements of BDD-TEM electrodes

ESI 3: Additional surface characterization data

ESI 4: Additional XPS data

ESI 5: Contact angle measurements

ESI 6: Electrochemical characterization

ESI 7: EELS data

ESI 8: MnO_2 crystallization d-spacing data

ESI 9: TEM grid temperature stability in air

References

ESI 1: Troubleshooting guide

Some common issues experienced when fabricating BDD-TEM electrodes are given in Table S1, with suggestions on how to avoid/circumvent these.

Table S1. BDD-TEM electrode fabrication troubleshooting guide.

Problem	Indicated by	Solution
Electron beam transparent areas around hole edge have snapped off during use	Very straight edges around hole as observed by EM, struggling to find areas thin enough to collect high quality TEM images	1. Use other thin areas in alternative locations around hole edge<br>2. Use a low angle (3-4°) PIPS clean for a short amount of time (~10 mins) to further polish BDD at hole edge. If unsuccessful after e.g., 30 – 60 mins:<br>3. Mill a new grid
Electron beam transparent area doesn't extend very far from hole edge	Often indicated by large central hole, difficult to find good locations for high quality TEM imaging	1. Use a low angle (3-4°) PIPS clean for a short amount of time (~10 mins) to further polish BDD at hole edge. If unsuccessful after e.g., 30 – 60 mins:<br>2. Mill new grid
Laser roughened area snaps off electrode along laser line	The contact is a fracture point, the grid will be in two pieces	1. Minimize number of laser roughening treatments where possible, use another part of the electrode outside edge to roughen for contacting<br>2. Use a C ink contact, where laser roughening is required once only<br>3. Take care when handling to avoid snapping the contact
Au NP contamination of BDD-TEM grid caused by incomplete masking during sputtering of Ti/Au contact, or dissolution of Au contact during long term experiments in acid solutions	Au NPs near hole seen in TEM. Contamination can be verified via EDX analysis or EELS in TEM	1. Acid clean electrode to remove Au contamination and use C ink contact instead, especially in long term experiments
C contamination, from prolonged TEM imaging	A bright halo forming at the coastline, clearly amorphous and localized to imaged areas	1. Prevent formation in the first instance: store samples under vacuum desiccator where possible. Bake samples in a vacuum prior to imaging (e.g. 65 °C)

		2. Clean by electrochemical cycling in sulfuric acid (*e.g.* 0.5 M) into the OER window (*e.g.* 2 V *vs* Ag/AgCl ~30 minutes), check contamination removed (briefly) with TEM at suitable magnification. If not effective, a full acid clean is required and start again with the experiment
C contamination, from long term acid cycling experiments when using a C ink contact	Observable in TEM, a thin film appears to coat the sample, amorphous, and may change (be careful to avoid contaminating TEM) under electron beam	1. Minimize C ink contact area, and ensure the contact is as far from solution meniscus as possible. If the contact does become wetted by solution, remove, and allow to dry before dipping a second time<br>2. A larger film from long term AST experiments should be removed via full acid clean, steps should be taken to minimize applied C ink area and keep well away from solution meniscus

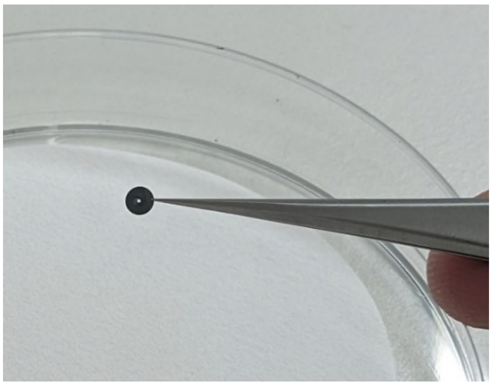

Figure S1: Photograph of a resulting BDD-TEM electrode (3 mm in diameter) being handled with tweezers. The central hole can also be seen.

ESI 2: Uncompensated resistance measurements of BDD-TEM electrodes

In a potential range where no faradaic reactions occur, the electrode can be treated as ideally polarizable. Under these conditions, the current flowing, *i*, in response to a potential pulse, *ΔE* is described by eq. S1:[1,2]

$$i(t) = \frac{\Delta E}{R_u} e^{-t/R_u C} \qquad \text{(S1)}$$

where R_u is the uncompensated resistance (Ω), *t* is the total time (s), and *C* is the capacitance (F). R_u measurements were made using *ΔE* of 0.1 V (0.20 V to 0.30 V vs Ag/AgCl) for 5 ms in 0.1 M KNO_3. Five separate pulses were recorded for each electrode contact, and the ***i*-*t*** response recorded, Figure S2. Each pulse was fitted according to eq. S2 following the Levenberg Marquardit iteration algorithm:

$$y = ae^{bx} \qquad \text{(S2)}$$

where *b* is equal to $-1/R_uC$. R_u can then be calculated via eq. S3, where *a* is the pre-exponential term from the fitted function (eq. S2):

$$R_u = \frac{\Delta E}{a} \qquad \text{(S3)}$$

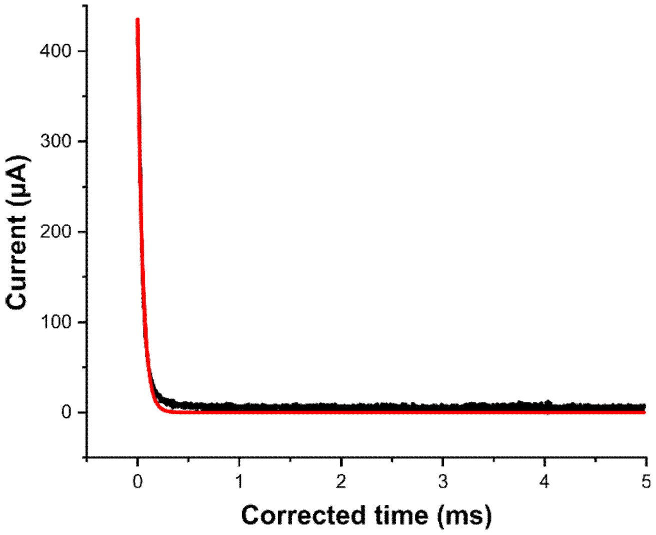

Figure S2: Example response (black) and corresponding exponential fit (red) of a BDD-TEM electrode with C ink contact.

Table S2 contains the experimentally determined *a* and R_u values for two BDD-TEM electrodes, one with a Ti/Au electrical contact and the other with a conductive carbon ink contact.

Table S2: Fitted *a* parameter and the corresponding R_u values from 5 chronoamperometric pulses, the average R_u, and the standard deviation.

	a ($\times 10^{-4}$)	R_u (Ω)	Average R_u (Ω)	Standard Deviation (Ω)
**BDD-TEM Ti/Au contact**	4.35	230	229	1.2
	4.35	230		
	4.38	229		
	4.41	227		
	4.35	230		
**BDD-TEM C ink contact**	2.83	354	346	3.8
	2.89	346		
	2.90	346		
	2.90	345		
	2.90	345		

ESI 3: Additional surface characterization data

WLI measurements were also recorded (Fig. S3) to investigate the topography of the surface. The surface roughness was also measured (Fig. S3b) over consecutive areas (20 × 40 µm) from the hole edge (hole is colored grey) to the electrode edge. An increasing surface roughness was observed (Fig. 3bii) from ~200 nm RMS at the edge of the hole to ~300 nm RMS ca. 200 µm away from the hole.

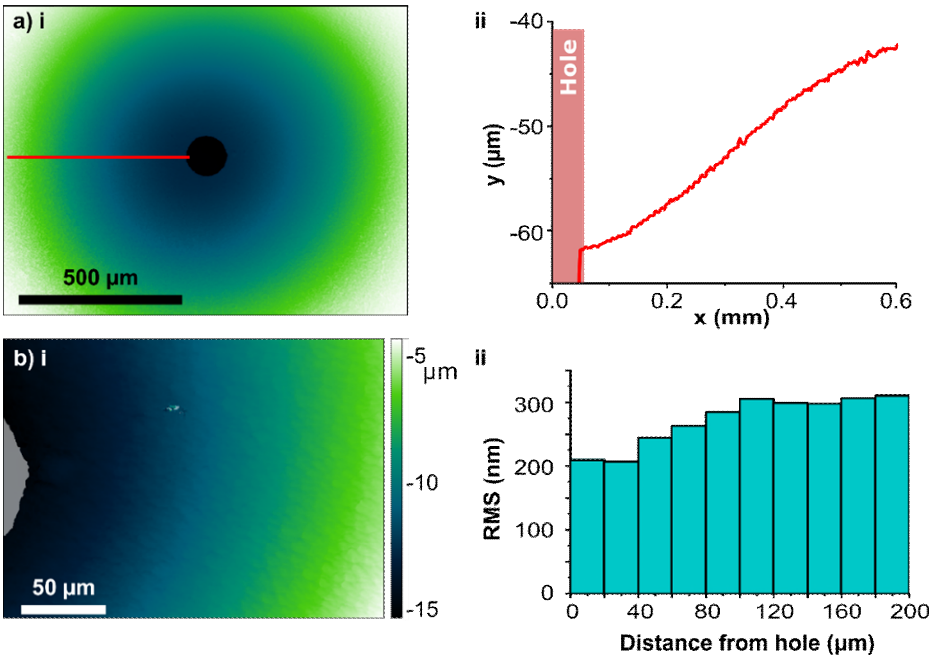

Figure S3. WLI data of a BDD-TEM electrode, (a) at lower resolution, showing (i) WLI image and (ii) a corresponding line profile (red line) of the surface, where *y* is the vertical sample height and *x* is the horizontal distance. (b) at higher resolution, showing (i) WLI image and (ii) corresponding surface roughness, reported as RMS values, calculated using Gwyddion 2.5.2.[3]

Fig. S4 shows (a) STEM image and (b) AFM topography data collected on a BDD-TEM electrode that was produced using a Gatan PIPS II ion mill. The substrate has an RMS roughness of 15 nm for the area shown in Fig S4b. The RMS roughness over the line profile shown in Fig. S4b is 4.4 nm (Fig. S4c). The data shown indicates the PIPS II milling system creates a comparable BDD-TEM electrode to the PIPS I system.

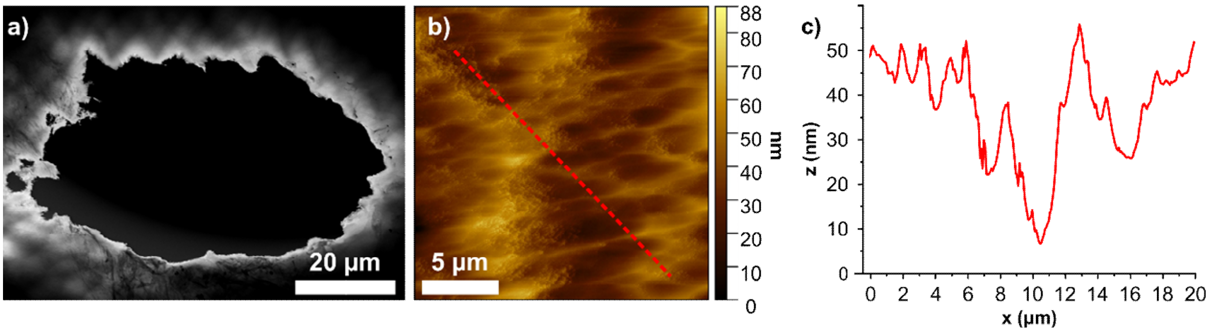

Figure S4: Characterization of PIPS II milled BDD-TEM electrode. (a) FE-SEM at 20 kV using the (S)TEM detector. (b) 20 × 20 µm AFM topography data and c) corresponding line profile of the surface, where *z* is the measured height and *x* is the position across the red line marked in (b). The AFM image was recorded very close to the hole edge.

ESI 4: Additional XPS data

XPS was used to examine the surface chemistry of an un-milled, mechanically polished (and acid cleaned) BDD surface (BDD control), compared with the ion milled/polished (and acid cleaned) BDD-TEM electrode. The C1s fittings for both samples are given in Table S3. Binding energies have been considered relative to the assigned sp^3 C-C/C-H peak for each electrode.

Table S3. C 1*s* fittings of BDD control and BDD-TEM expressed as percentages of the total fitted envelope.

	**Assigned functionality**	**Absolute binding energy (eV)**	**Binding energy shift relative to sp^3 peak (eV)**	**Relative composition of fitted envelope (%)**
**BDD control 90° (Fig. 4a)**	sp^2 C – C	284.1	-0.7	20.8
	sp^3 C – C / C – H	284.8	0	71.6
	C – O	286.4	+1.6	7.0
	π – π*	290.7	+5.9	0.6
**BDD control 30° (Fig. 4b)**	sp^2 C – C	284.0	-0.8	9.3
	sp^3 C – C / C – H	284.8	0	77.7
	C – O	286.2	+1.4	12.8
	π – π*	290.5	+5.7	0.3
**BDD TEM 90° (Fig. 4c)**	sp^2 C – C	284.8	-0.9	10.2
	sp^3 C – C / C – H	285.7	0	77.2
	C – O	286.9	+1.2	12.4
	π – π*	291.5	+5.8	0.3

The C 1*s* spectra of a commercial TEM grid, an amorphous C film floated onto an Au support mesh (Agar Scientific), was acquired following the same experimental procedure as the BDD control sample. Figure S5 shows the fitted C1s spectra, and Table S3 shows the relative contributions of each peak expressed as percentages of the fitted envelope. It is clear from the C 1*s* fitting that this commercial grid has a high sp^2 carbon content (as expected), in this case 62%, and a much lower sp^3 carbon content at 27%. The contributions from C = O and O = C – O and π – π* are more pronounced on this sample (2% of the envelope each), indicating different surface functionality when compared to BDD.

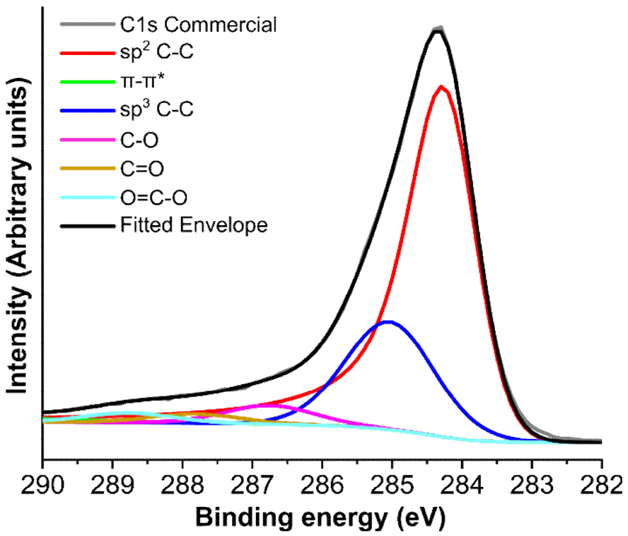

Figure S5: Fitted C1s spectra of commercial amorphous sp^2 bonded carbon film on an Au support mesh.

Table S4: C1s fittings of a commercial amorphous sp^2 carbon film on an Au support mesh expressed as percentages of the total fitted envelope.

	**Assigned functionality**	**Absolute binding energy (eV)**	**Relative composition of fitted envelope (%)**
**carbon TEM grid**	sp^2 C – C	284.3	62.0
	sp^3 C – C / C – H	285.0	26.9
	C – O	286.8	4.6
	C = O	287.8	2.3
	O = C – O	288.8	2.4
	π – π *	290.7	1.9

ESI 5: Contact angle measurements

Contact angle measurements were recorded (in triplicate) to compare the hydrophobicity and wetting of a BDD-TEM electrode vs a commercial amorphous carbon coated TEM substrate, C/Au TEM (Fig. S6). The average contact angles measured were 62.2 ± 0.5° and 83.6 ± 1.1° for the BDD and (amorphous) carbon substrates, respectively.

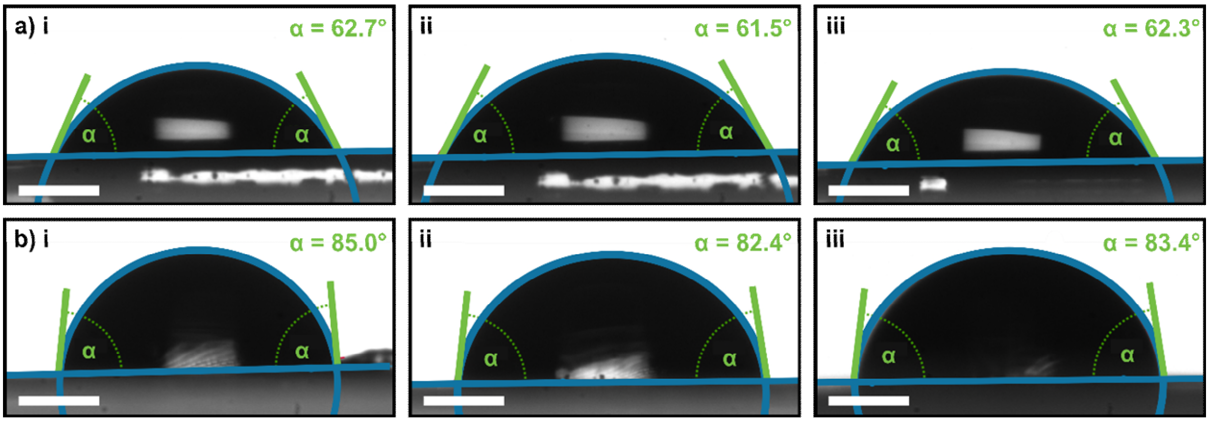

Figure S6. Triplicate repeat contact angle measurements of a 50 µL water droplet on (a) a BDD-TEM electrode (sp^3 bonded carbon) and (b) a C/Au commercial TEM grid (62% sp^2 bonded carbon).

ESI 6: Electrochemical Characterization

The solvent windows for both samples in 0.1 M KNO_3 at 0.1 V s^{-1} were wide and featureless (Fig. S7a), with values of 3.18 and 3.54 V (for a given geometric current density of ±0.4 mA cm^{-2}) for the BDD-TEM and BDD control electrodes, respectively. To calculate the electrochemical capacitance, *C*, the voltage window was decreased to 0 V ± 0.1 V (Fig. S7b) and equation S4 was used:

$$C = \frac{i_{av}}{\upsilon A} \quad \text{(S4)}$$

where i_{av} is the average current magnitude at 0 V from the forward and reverse sweep, v is the scan rate (0.1 V s^{-1}) and A is the geometric electrode area. Capacitance values of 5.3 and 4.3 µF cm^{-2} for the ion milled/polished and mechanically polished electrodes, respectively, were measured. For mechanically polished CVD-grown BDD a C of ≤10 µF cm^{-2} is typical (acquired using digital staircase CV).[47] The one-electron reduction of $Ru(NH_3)_6^{3+}$ was also studied by CV (Fig. S7c). For the mechanically polished electrode, a peak-to-peak separation, ΔE_p, of 68 mV was measured, compared to 70 mV, for the ion milled/polished electrode. These responses are close to reversible.

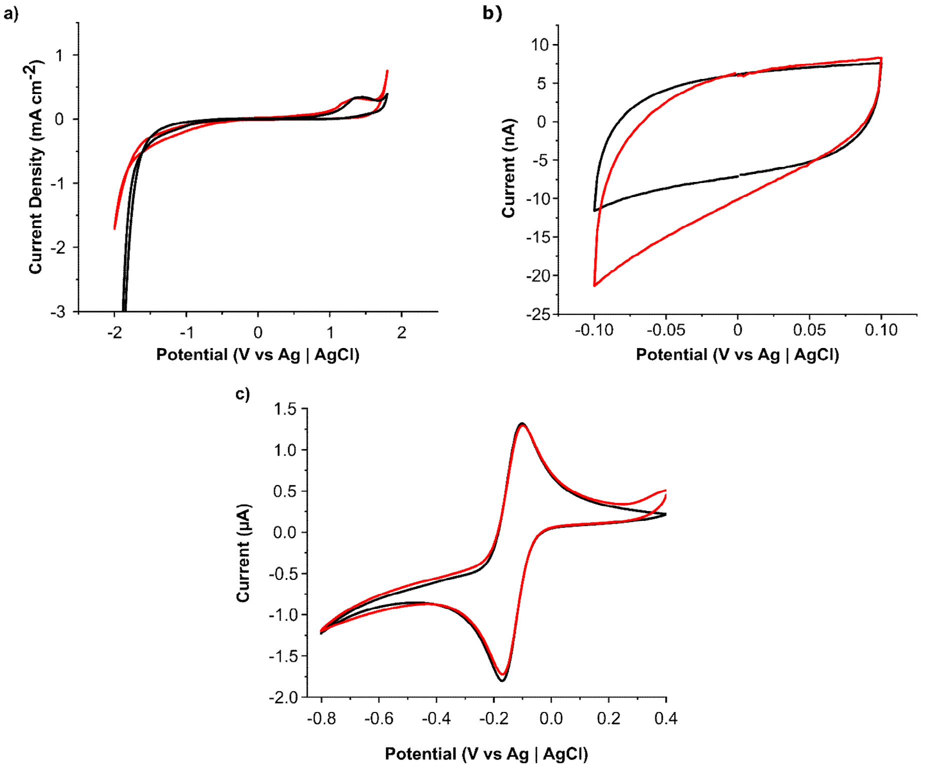

Figure S7. CVs recorded in 0.1 M KNO_3 at a scan rate of 0.1 V s^{-1} on a PIPS milled/polished BDD electrode (red) and a mechanically polished BDD surface (black). Shown are (a) solvent windows, (b) typical capacitance curves recorded to calculate capacitance, and (c) electrode response in 1 mM $Ru(NH_3)_6^{3+/2+}$ (0.1 M KNO_3), third scan CVs shown.

ESI 7: EELS data

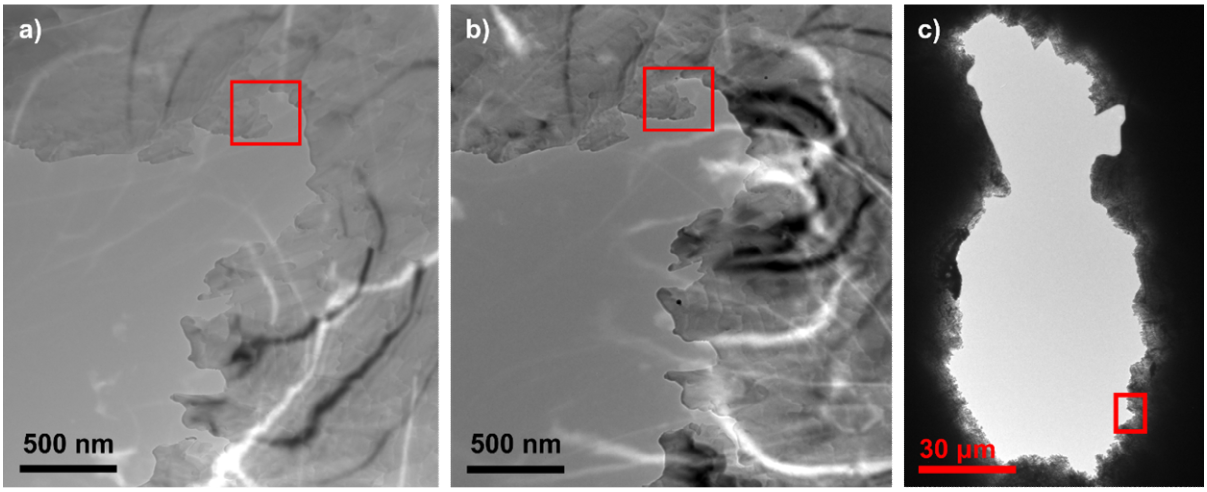

Figure S8. Diffraction images of the hole edge (a) before and (b) after electrochemical stability testing. Red rectangles indicate areas shown in Fig. 4 aii,iii (main text). (c) Diffraction image of hole edge at much lower magnification, red rectangle indicates the areas in Fig. S8a,b. The coastline of the hole edge has clearly recognizable features which allows IL-TEM imaging.

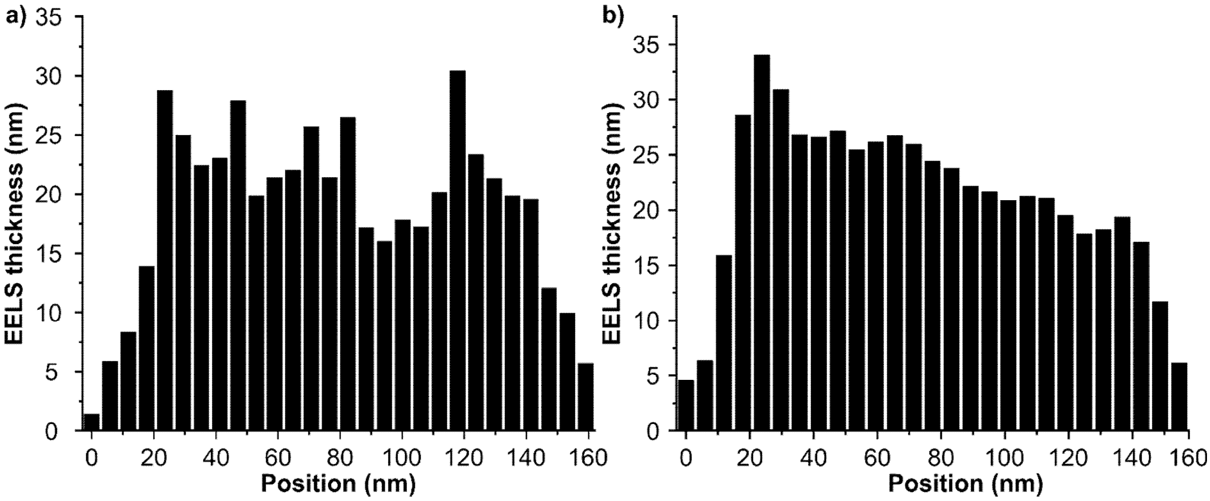

Figure S9. Representative BDD-TEM thickness profiles extracted over a 160 nm line profile (28 measurements) from 2D EELS spectra, acquired over the identical location areas highlighted in Figs. 4aii and 4aiii, (a) before electrochemical cycling and (b) after electrochemical cycling.

ESI 8: MnO_2 crystallization d-spacing data

The diffraction patterns shown in Figs. 5 bii and cii were taken with camera lengths of 150 and 300 mm, respectively. The diffraction pattern in Fig. S10a was obtained after heating the MnO_2-BDD TEM electrode to 200°C and then allowing to cool; the MnO_2 is amorphous, but diffraction features corresponding to the BDD support are clearly visible, for example in the region inside the blue box. Whilst the BDD surface is predominantly (110) textured, in some regions twinning of the surface is evident resulting in non (110) orientations. The zone axis at the bottom left of this pattern is identified as [211] and the systematic rows of spots visible inside the blue box are thus 111, 222, 333, etc. Knowing the lattice constant of diamond, $a_{diamond}$ = 3.567Å, these spots can be used to calibrate the camera length, (d^*_{111} = 165 pixels = $\sqrt{3}/a_{diamond}$ = 0.4856Å^{-1}). Fig. S10b shows a pattern obtained after MnO_2 crystallisation (induced by heating to 400°C), converted from polar to Cartesian coordinates. This allows accurate measurement of d^*-spacings, indicated with red arrows, observed at 0.462, 0.521, 0.599, 0.684 and 0.708 Å^{-1}. These are a good match with those reported in the literature for γ-MnO_2 (Table S5).[3]

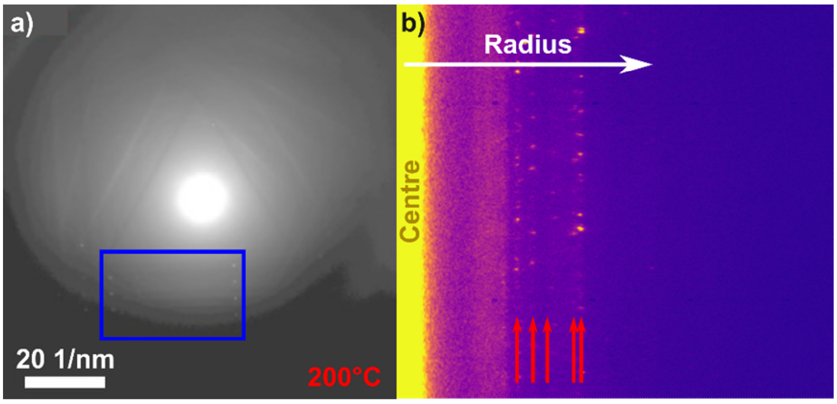

Figure S10. (a) Diffraction pattern of electrodeposited MnO_2 on a BDD-TEM electrode after heating to 200°C. Blue rectangle indicates location of the BDD diffraction spots used to calibrate image scale. (b) Cartesian depiction of the diffraction pattern shown in Fig. 5cii. Red arrows indicate d^*-spacings.

Table S5. Comparison between experimentally obtained *d**-spacing values for crystalline γ-MnO_2, obtained by thermal *in-situ* TEM annealing of electrodeposited amorphous MnO_2, and previously reported *d** for electrochemically active γ-MnO_2 (#62 *Pnam*, *a* = 9.32Å, *b* = 4.46Å, *c* = 2.85Å, reference 3).

***d** (Å) experimental ±0.01Å**	***d** (Å) from ref 3**	**hkl**	**Nominal I/I_0 (%)**
0.410	0.413	111	27
0.462	0.468	211	36
0.521	0.526	311	15
0.599	0.608	221	61
0.654	0.679	511	39
0.708	0.702	002	100

ESI 9: TEM grid temperature stability in air

Both BDD-TEM and commercial C/Cu grids were heated to elevated temperatures of 200°C and 400°C in air (Fig. S11). For the BDD-TEM grids no visual changes were observed (Fig. S11a), and the same grid could be used for all the heating studies. A new C/Cu commercial TEM grid was used for each temperature as the grid was visibly damaged after heating (Fig. S11b), including holes in the carbon film. After heating for 4 hours at 400°C, most of the squares in the TEM grid are devoid of C film (Fig. S11biii).

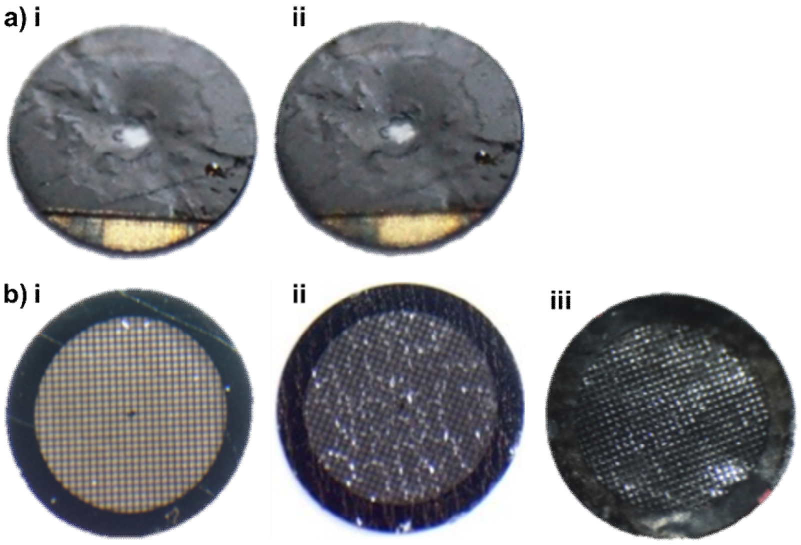

Figure S11. Photographs of (a) a BDD-TEM electrode (i) before heating in air and (ii) after heating in air to 200°C and then 400°C for 4 hours at each temperature, and b) a C/Cu commercial TEM grid (i) before heating in air and (ii-iii) after heating in air for 4 hours to ii) 200°C, and iii) 400°C. A fresh C/Cu grid was used for each annealing temperature due to induced damage in each heating experiment. The same BDD-TEM electrode was used throughout with annealing temperature increasing from lowest to highest. Note all TEM grids are 3 mm in diameter.

References

1. S. Cobb, J. Macpherson, *Anal. Chem.*, 2019, **91**, 7935 – 7942

2. J. C. Myland, K. B. Oldham, Uncompensated Resistance. 1. The Effect of Cell Geometry. *Anal. Chem.,* 2000, 72 (17), 3972 – 3980.

3. Yu.D. Kondrashev, A.I. Zaslavskii, *Izvestiya Akademii Nauk SSSR, Seriya Fizicheskaya,* 1951, **15**, 179-186.
